# Alcohol and violence in 2017 National Football League Super Bowl commercials

**DOI:** 10.15171/hpp.2017.29

**Published:** 2017-06-14

**Authors:** Sarah A. MacLean, Corey H. Basch, Philip Garcia

**Affiliations:** ^1^Department of Epidemiology, Mailman School of Public Health, Columbia University, New York, NY, USA; ^2^Department of Public Health, William Paterson University, Wayne, NJ, USA

**Keywords:** Advertising, Alcohol, Commercial, Super Bowl, Risky behavior, Television, Violence

## Abstract

**Background: ** The National Football League (NFL) Super Bowl is a widely-viewed sports event and the commercials are especially popular among viewers. Previous research has demonstrated risky health behaviors in advertisements aired during sporting events. The purpose of this study was to analyze the content of the advertisements aired during the 2017 NFL Super Bowl.

**Methods:** This cross-sectional study involved examining the content of all commercials, with an emphasis on health-compromising behaviors. The themes and highlights of the advertisements were analyzed based on whether there was a reference to alcohol or violence.

**Results: ** A total of 103 unique commercials were analyzed. The most common themes were humor (n=43), happiness (n=25), innovation (n=25), and enjoyment or relaxation (n=25).Alcohol was referenced in 13 (12.6%, 95% CI 7.5%, 20.4%) of the commercials. Advertisements with alcohol references were more likely to contain the themes of partying (odds ratio [OR]:16.2, 95% CI 1.4-193.4, P=0.041) and enjoyment or relaxation (OR: 4.7, 95% CI 1.4-15.6,P=0.014). There were 24 commercials with references to violence and these were more likely tobe promoting a movie (OR: 5.4, 95% CI 3.5-8.2, P<0.001) or television program (OR: 8.9,95% CI 2.6-30.26, P<0.001).

**Conclusion:** Parents should consider whether it is appropriate for their children to watch a concentrated number of intense images containing references to alcohol and violence during this popular sporting event.

## Introduction

The 2017 National Football League (NFL) Super Bowl Championship was viewed by an estimated 11.3 million people, placing it fourth among the most-viewed television programs in US history.^1^ The high cost of advertisements during the Super Bowl, estimated at $3million per 30-second ad in 2009, reflects the importance of the event’s high viewership.^2^ Given the high price, viewers have come to expect interesting commercials; in fact, more people say the commercials are their “favorite thing about the Super Bowl” than the game itself.^3^Academics have highlighted the importance of Super Bowl commercials as the “game within the game.”^4^

In 2011, $778 million was spent on marketing alcohol on US television programs.^5^ The alcohol industry has self-regulatory codes intended to limit alcohol advertising to underage consumers,^5^ yet programs such as sporting events are viewed by people of a wide age range. An estimated 30.2% of teens and 21.8% of kids age 2-11 watched the 2014 NFL Super Bowl.^6^ Given that underage drinking is a public health problem in the United States, with 20.3% of people ages 12-20 reporting drinking alcohol in the past month,^7^ the advertisement of alcohol during widely-viewed sporting events such as the NFL Super Bowl is concerning.

Violence is another common theme used in the media in the United States. Anderson and Swing cite media violence exposure as a risk factor for aggression.^8^ Children who are exposed to violence experience heightened stress and, as a result, adverse health reactions.^9^ Furthermore, children who witness interpersonal violence among adults have demonstrated cognitive issues later in life.^10^ Despite the risks, violence is still a recurring theme in advertising. Producers may create violent content in an attempt to generate revenue.^11^ Advertisers frequently utilize media that contain violence due to the belief that this content draws larger audiences. Among the 100 highest rated TV programs, 100 top-grossing films, and 50 top-selling video games from 2009 through 2014, 48% were specifically rated for violent content.^11^ People in the 18-34 year old age group are most drawn to violence in the media and also have the most disposable income. Advertisers believe that younger adults are more easily influenced by commercials than older adults with more established purchasing habits.^11^ There is concern as to whether the health or decisions of viewers in the 18-34 age group are affected by these violent advertisements.

There has been limited research on the content of commercials aired during the NFL Super Bowl and particularly how it relates to public health. One study of all major American competitive events from 2001-2002 found the NFL Super Bowl to have the highest percentage of commercials with unsafe or violent content.^12^ A study of commercials during the 2003 NFL Super Bowl found that 15 of the 55 contained beer or malt liquor.^13^A previous analysis of commercials during the 2015 Super Bowl demonstrated that wild driving behavior, violent or sexual content, and substance use were common.^14^ Based on this previous research and the public health implications of advertising risky behaviors, we conducted this study to describe content related to alcohol and violence in the advertisements aired during the 2017 NFL Super Bowl.

## Material and Methods

### Study design and variables

A coding sheet was created based on several sources described below. First, basic information regarding the commercial was taken. This included the exact time of airing, length in time, position of block of commercials, total number of commercials in that block, and the overall product or service the commercial focused on. The coder then indicated whether the commercial was specifically advertising an alcohol product. The type of alcohol featured was coded as beer, wine, wine cooler, spirits, mixed drinks, or other. The use of alcohol in the commercial was as follows: actors drinking; drinking is implied; other reference; or brand appearance. Food featured, either consumed or as a product placement, was also specified as: snack or junk food; food from restaurant chain; fast food; cultural foods; fine dining; and other. Demographic information of either the celebrities used in the commercial or actors was included as age range, race, and gender.

Themes of each video were dichotomously coded as “yes” indicating presence of the theme, or “no” when theme was not present. The themes were set from other studies which analyzed advertisements for alcohol.^14,15^ They are as follows: partying; having fun with friends; being cool; enjoyment/relaxation; happiness; having a free spirit; being masculine or manly; being feminine; sports, either competitive or leisure; being an innovator or seeking status; humor; tradition; household/lifestyle; violence; political; artistic/imagery; and luxury.

Behaviors of interest were noted that could have compounding effects when drinking alcohol. These elements were violence, types of foods, if any were consumed, automobile use, sexual behaviors, comedy/humor use, and other risky behaviors. Violence in the commercials was further analyzed in the coding sheet again using dichotomous variables “yes” and “no.” If the commercial had violence as a theme, it was identified whether or not there was use of explosions, guns, or physical harm using either hands or objects.

The presence of popular selling points was noted. These selling points, adapted from Resnick and Stern,^16^ were: components/ingredients; price/value; guarantee/warrantee; safety mentioned; company sponsored research mentioned; new ideas/concepts introduced or mentioned; independent research mentioned; and special offers mentioned. Highlighting the quality of the product, packaging, nutrition, taste, performance, or availability was dichotomously coded as “yes” or “no.”

### Study size

In order to provide as complete a description of commercials as possible, we included all commercials aired during the event rather than sampling a subset.

### Statistical analyses

SPSS (version 24.0 Armonk, NY: IBM Corp) was used to calculate descriptive statistics and run statistical tests. Odds ratios (OR) with 95% confidence intervals (95% CI) and Fisher’s exact tests of association were used to compare commercials with and without references to alcohol and violence. Chi-square tests were used in place of Fisher’s exact tests when all expected cell counts were greater than 5. We considered results of *P* < 0.05 to be statistically significant.

## Results

We analyzed 103 unique commercials, the majority of which were 25-31 seconds in length (Table 1). Most portrayed people in the 21-30, 31-40, and 41-50 age ranges. The most common elements highlighted in the commercials were quality (n = 41), new ideas (n = 18), and taste (n = 16). The most common themes were humor (n = 43), happiness (n = 25), innovation (n = 25), enjoyment or relaxation (n = 25), and violence or action (n = 24).

Alcohol was referenced in 13 (12.6%) advertisements. In ten of the commercials, drinking alcohol was implied rather than explicitly viewed. Commercials containing references to alcohol were significantly more likely to contain the themes of partying (OR: 16.2, 95% CI: 1.4-193.4, *P* = 0.041), enjoyment or relaxation (OR: 4.7, 95% CI: 1.4-15.6, *P* = 0.014), luxury (OR: 5.3, 95% CI: 1.3-21.5, *P* = 0.031), and tradition (OR: 16.2, 95% CI: 1.4-193.4, *P* = 0.041) than commercials that did not reference alcohol (Table 2).

Twenty-four commercials (23.3%) contained references to violence and action. Examples included physically hurting others with hands or objects. Commercials containing violence were 5.4 (95% CI: 3.5-8.2, *P* < 0.001) times as likely to be used as a movie promotion and 8.9 (95% CI: 2.6-30.3, *P* < 0.001) times as likely to be used as a TV show promotion.

## Discussion

In this study, we found evidence of risky health behaviors in many of the commercials aired during the 2017 NFL Super Bowl. Given the wide viewership of this event and the popularity of the commercials, this is an area of public health concern. Advertising has the demonstrated power to negatively influence consumers’ views of themselves and societal norms.^17^ This is especially true for children, as the American Academy of Pediatrics has found a strong causal relationship between the consumption of media and the demonstration of comparable behaviors among children.^18^

Nearly one quarter (23%) of the commercials aired during the 2017 NFL Super Bowl contained violence or action scenes. This percentage is down from 29% during the 2015 NFL Super Bowl,^14^ but nevertheless is a substantial percentage of the commercials aired during the program. The Federal Communications Commission does not regulate the broadcast of violent programming on network television.^19^ Previous studies have found that violent and age-inappropriate advertisements are routinely aired during major sporting events, including widely-viewed playoff games of Major League Baseball and the NFL.^12,20^

Commercials containing violent images were significantly more likely to be intended to promote a movie or television show. This suggests that these programs are also sources of messages related to violence. While it is easier for parents to prevent their children from viewing full television shows or movies, it may be more difficult to prevent young viewers from watching these 30-second commercials with concentrated images of intense violence. Advertisements containing violence were also less likely to highlight positive themes such as happiness, innovation, and fun with friends.

About 13% of commercials aired during the NFL Super Bowl contained images that promote the sale of alcohol. This is up from less than 5% during the 2015 Super Bowl.^14^ Alcohol consumption and abuse are common in the United States. In 2011, US alcohol suppliers received $60 billion in gross revenue.^21^ Among people 18 or older in the U.S., 70.1% report drinking in the past year.^7^ Underage drinking is also a major concern in the United States, as 20.3% of people ages 12-20 report drinking alcohol in the past month.^7^ Previous research has demonstrated that viewing advertisements for alcohol not only increases the likelihood that adolescents will start to drink but also increases the frequency at which baseline drinkers consume alcohol.^22^ This relationship between alcohol advertising and consumption has also been demonstrated in the specific realm of sports sponsorship by alcohol brands.^23^

Commercials referencing alcohol were significantly more likely to have partying as a dominant theme. Previous studies have found partying to be a dominant theme in 42% of alcohol advertisements aired on US television.^15^ Our results support the assertion of Morgenstern et al that future research is necessary to understand if exposure to partying themes predicts alcohol use among young viewers.^15^ This is particularly important given the rise in social media use and the demonstration that alcohol brands use sports to engage with consumers on social platforms.^24^

This study is limited in that it was cross-sectional and data were only collected at a single time point. Furthermore, the actual viewership of the NFL Super Bowl broadcast can only be estimated. Many people watch the game in large groups, making it difficult to capture viewing statistics. Future studies should examine the advertisements of other television programs with larger viewership, such as awards shows and other large sporting events.

Despite these limitations, the results of this study demonstrate the opportunity for health promoters to use this popular sporting program as a health education tool. Public health advocates can focus on key factors, such as enhancing consumers’ ability to identify marketing tactics used to promote alcohol consumption and the negative consequences of using violence in marketing. In particular, parents should be educated about the content of commercials and the possible effects (such as mirroring violent behavior or brand recognition of alcohol) on their children if they view this type of content.

## Conclusion

On the surface, the NFL Super Bowl is a football game, but a deeper analysis of the content shown during commercial breaks demonstrates that it is a program that may not be appropriate for all viewers. The advertisements, which for many people are just as important as the game, often contain messages related to risky health behaviors. Parents should consider whether it is appropriate for their children to watch a concentrated number of intense images containing references to alcohol and violence during this event.

## Ethical approval

This study was considered exempt from review by the Institutional Review Board (IRB) at William Paterson University.

## Competing interests

The authors report no competing interests.

## Acknowledgements

No funding was obtained for this study.

**Table 1:** Elements of commercials analyzed

	**N = 103**	**Percent**
Length (s)		
10-15	21	20.4
25-31	59	57.3
35-45	4	3.9
60-90	19	18.4
Alcohol reference		
Any	13	12.6
Actors drinking	5	4.9
Implied	10	9.7
Brand appears	4	3.9
Other reference	6	5.8
Type of alcohol		
Beer	8	7.8
Wine/wine cooler	8	7.8
Spirits/mixed drinks	7	6.8
Foods		
Snack	6	5.6
Restaurant chain	6	5.8
Fast food	3	2.9
Fine dining	1	1.0
Highlights		
Ingredients	3	2.9
Price value	8	7.8
Guarantees or warranties	5	4.9
Safety	4	3.9
Research	0	0.0
New ideas or concepts	18	17.5
Quality of product	41	39.8
Packaging	3	2.9
Nutrition	4	3.9
Taste	16	15.5
Special offers	5	4.9
Availability	10	9.7
Performance	1	1.0
Celebrity appearance	29	28.2
Genders shown		
Males only	21	20.4
Females only	0	0.0
Both	76	73.8
Neither (no people)	6	5.8
Ages portrayed (y)		
Under 21	29	28.2
21-30	58	56.3
31-40	80	77.7
41-50	53	51.5
50+	30	29.1
Themes & elements		
Partying	3	2.9
Fun with Friends	13	12.6
Being Cool	4	3.9
Enjoyment/Relaxation	25	24.3
Happiness	27	26.2
Having a free spirit	4	3.9
Manly	4	3.9
Women	7	6.8
Sports	19	18.4
Comedy/humor	43	41.7
Innovation/status	25	24.3
Household/lifestyle	8	7.8
Political	3	2.9
Art/Imagery	11	10.7
Luxury	11	10.7
Tradition	3	2.9
Love	10	9.7
Violence/action	24	23.3
Explosions/guns	17	16.5
Movie promotion	6	5.8
TV show promotion	14	13.6
Sexual use/hints	11	10.7
Health promotion	6	5.8
Catch phrase	19	18.4
Explosions/guns	17	16.5

**Table 2:** Associations of themes with presence of alcohol or violence/action in commercial

**Themes & Elements**	**N**	**%**	**Alcohol Reference**			**Violence/Action**		
			**Yes**	**No**	***P***	**Yes**	**No**	***P***
Total	103	100	13	90		24	79	
Partying	3	2.9	2	1	**0.041**	0	3	1.000
Fun with friends	13	12.6	4	9	0.058	0	13	**0.035**
Being cool	4	3.9	0	4	1.000	0	4	0.571
Enjoyment/relaxation	25	24.3	7	18	**0.014**	2	23	0.055^a^
Happiness	27	26.2	5	22	0.318^a^	0	27	**<0.001** ^a^
Having a free spirit	4	3.9	0	4	1.000	0	4	0.571
Manly	4	3.9	2	2	0.077	2	2	0.231
Women	7	6.8	1	6	1.000	4	3	0.050
Sports	19	18.4	3	16	0.703	0	19	**0.006** ^a^
Comedy/humor	43	41.7	5	38	1.000	5	38	**0.019** ^a^
Innovation/status	25	24.3	2	23	0.730	1	24	**0.007**
Household/lifestyle	8	7.8	0	8	0.591	0	8	0.193
Political	3	2.9	0	3	1.000	0	3	1.000
Art/imagery	11	10.7	1	10	1.000	5	6	0.123
Luxury	11	10.7	4	7	**0.031**	2	9	1.000
Tradition	3	2.9	2	1	**0.041**	1	2	0.553
Love	10	9.7	2	8	0.611	1	9	0.446
Violence/action	24	23.3	8	18	0.072	–	–	–
Explosions/guns	17	16.5	4	13	0.221	17	0	**<0.001**
Movie promotion	6	5.8	2	4	0.165	6	0	**<0.001**
TV show promotion	14	13.6	1	13	1.000	9	5	**<0.001**
Sexual use/hints	11	10.7	2	9	0.627	4	7	0.277
Health promotion	6	5.8	1	5	0.565	0	6	0.332
Catch phrase	19	18.4	0	19	0.119	0	19	**0.006**

^a^Chi-square test used instead of Fisher’s exact test
